# Defects in Silicene: Vacancy Clusters, Extended Line Defects, and Di-adatoms

**DOI:** 10.1038/srep07881

**Published:** 2015-01-26

**Authors:** Shuang Li, Yifeng Wu, Yi Tu, Yonghui Wang, Tong Jiang, Wei Liu, Yonghao Zhao

**Affiliations:** 1 Nano Structural Materials Center, School of Materials Science and Engineering, Nanjing University of Science and Technology, Nanjing 210094, Jiangsu, China

## Abstract

Defects are almost inevitable during the fabrication process, and their existence strongly affects thermodynamic and (opto)electronic properties of two-dimensional materials. Very recent experiments have provided clear evidence for the presence of larger multi-vacancies in silicene, but their structure, stability, and formation mechanism remain largely unexplored. Here, we present a detailed theoretical study of silicene monolayer containing three types of defects: vacancy clusters, extended line defects (ELDs), and di-adatoms. First-principles calculations, along with *ab initio* molecular dynamics simulations, revealed the coalescence tendency of small defects and formation of highly stable vacancy clusters. The 5|8|5 ELD – the most favorable extended defect in both graphene and silicene sheets – is found to be easier to form in the latter case due to the mixed sp^2^/sp^3^ hybridization of silicon. In addition, hybrid functional calculations that contain part of the Hatree-Fock exchange energy demonstrated that the introduction of single and double silicon adatoms significantly enhances the stability of the system, and provides an effective approach on tuning the magnetic moment and band gap of silicene.

Silicene, a hexagonal mesh of silicon atoms, has attracted significant interest from both academia and electronics industry because it exhibits comparable electronic characteristics as graphene, *e.g.*, the existence of the Dirac cone and the realization of the quantum spin Hall effect^1^,^2^,^3^,^4^,^5^. Besides that, silicene could be synthesized and processed using mature semiconductor techniques, being more readily integrated into existing electronics than graphene. Unlike graphene, silicene forms a slightly buckled monolayer structure^6^,^7^ that allows more flexibility to tailor their band structures and functions^8^,^9^,^10^,^11^,^12^. Despite such single-layer silicon has been theoretically speculated for two decades^6^, the actual fabrication of silicene on many substrates, such as Ag(111)^13^,^14^, ZrB_2_(0001)^15^, (2 × 1)-reconstructed Au(110)^16^, and Ir(111) surfaces^17^, has been reported only very recently. The successful synthesis of silicene ribbons and sheets shows a promising application potential in nanoscale materials and devices^18^.

Defects are often the first concern in the real application of monolayer materials. Vacancy defects, which are readily induced by laser irradiation and electron beam, are almost inevitable in the fabrication and processing of monolayers, and sometimes, small defects are introduced purposively for specific applications^19^,^20^. For example, the creation and elimination of point defects have been shown to provide a powerful avenue to tune the local structure, thermal stability, and band gap of low-dimension materials^21^,^22^,^23^,^24^. In addition, the two dimensional materials with defects has been proposed to be a superior membrane for gas separation^25^,^26^.

Several theoretical works have concentrated on the structure and energetics of point defects in free-standing silicene. For example, Özçelik *et al.*^23^ studied the atomistic mechanisms in the self-healing of vacancy defects, and uncovered that the reconstruction process is driven by the energy gained through new Si-Si bond formation. Guo *et al.*^22^ systemically investigated the structures, formation energy, and migration behaviors of typical single vacancy and double vacancies. Berdiyorov and Peeters^27^ reported that vacancy defects include local structural changes and considerably reduce the thermal stability of silicene. These prior studies, generally focusing on the single, double, and triple defects in silicene, deepen one's understanding on the formation and diffusion mechanisms of vacancies in silicene. However, very recent experimental studies have provided strong evidence that a large number of defect clusters are present in the scanning tunneling microscope images, with more than three missing silicon atoms^28^. Although the presence of multi-atom vacancies is of fundamental importance for understanding the formation and operation of layered materials, their configurations, stabilities, and formation mechanisms are far from being fully comprehended.

In this contribution, we performed density-functional theory (DFT) calculations to investigate the reconstruction, coalescence, and diffusion behaviors of vacancy defects in free-standing silicene. The structure and energetic of vacancy clusters formed by removing 1–6 atoms were systematically considered, where upon coalescence occurs spontaneously to reduce dangling bonds on the edges. We then focused on five architectures of extended line defects (ELDs) embedded in silicene, showing that ELDs have lower formation energies compared to vacancy clusters, which arises from the reconstruction of periodic defects. Finally, we studied the adsorption and diffusion of silicon atoms on silicene layers, and uncovered that such adsorption process can enhance the stability of the system and open sizeable band gaps in silicene.

## Results

The formation energy per unit-cell involved in the reconstruction, *E_f_*, which indicates the stability of a defect in silicene, can be determined as
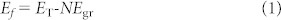
where *E*_T_ is the total energy of a silicene plane containing defects. *N* denotes the number of atoms in the unit cell, and *E*_gr_ is the energy per silicon atom in a pristine silicene sheet. Note that the magnitude of *E_f_* depends on the number of missing atoms, η, in the silicene sheet. In order to compare the relative stability of systems containing *different* numbers of vacancies, we further compute the formation energy *per missing atom*, *E_f_*′, which is defined as
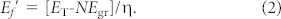


### Reconstruction and coalescence of vacancies in silicene

**Figure 1:**
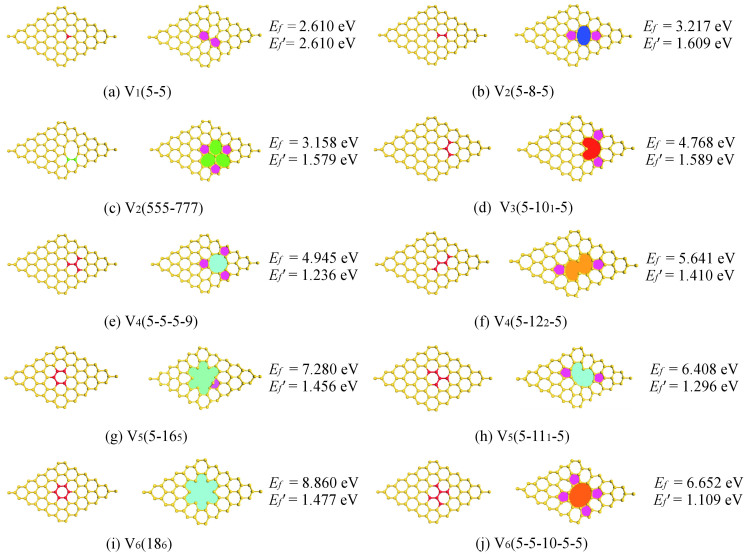
Silicene structures with one to six vacancies before (left) and after (right) full relaxation. The removed atoms and the rotated bond are shown in red and green, respectively. For each system, the numbers in the parentheses indicate nonhexagonal rings present in the system, where the subscript denotes the number of dangling bonds. For comparison purpose, the formation energy *E_f_* and the formation energy per missing atom *E_f_*′ are also shown in the figure.

We started by considering all possible configurations for the smaller vacancy clusters (1–3 missing atoms), and the most-likely configurations for the larger clusters (4–6 missing atoms). These vacancy defects were created through removing 1–6 silicon atoms from a pristine lattice. We noticed that even a single vacancy defect could lead to a significant local distortion of silicon's hexagonal arrangement (see Fig. 1a), in agreement with previous literature^22^. In addition, all considered structures shown in Fig. 1 have higher formation energies, thus less stable, than the pristine silicene.

Figures 1a–1d show the reconstructed silicene layer with 1–3 missing atoms. For single vacancy, we considered two reconstructed structures, V_1_(5-5) and V_1_(5-3)^22^,^23^, and found that V_1_(5-5) has a lower formation energy than V_1_(5-3) [2.61 *vs*. 3.24 eV]. In fact, our calculations show that V_1_(5-3) is a metastable structure and could transform to V_1_(5-5) by overcoming a barrier of 0.91 eV. Removing two neighboring silicon atoms forms double vacancies in silicene, as shown in Fig. 1b, no dangling bond appears in the reconstructed V_2_(5-8-5). The rotation of one of the bonds in the octagon of V_2_(5-8-5) transforms the defect into three pentagons and three heptagons, V_2_(5-5-5-7-7-7), which has lower formation energy than V_2_(5-8-5). The reconstructed structure after removing three atoms from the lattice is depicted in Fig. 1d, where two five-membered and a ten-membered (heart-shape) rings are formatted, V_3_(5-10_1_-5). In this case four dangling bonds are saturated, while one dangling bond still exists.

Figures 1e–1j illustrate the initial structures of silicene monolayer with 4–6 vacancies and their fully reconstructed counterparts. Apparently, more complicated defect configurations are formed upon the removal of more than three silicon atoms from the silicene sheet. The V_6_(18_6_) shown in Fig. 1i is found to be the most unfavorable structure, which is presumably because of the presence of the under-coordinated Si atoms. The same observation, *i.e.*, under-coordinated Si atoms destabilizes systems, is also applied for the V_3_(5-10_1_-5), V_4_(5-12_2_-5), V_5_(5-16_5_), and V_5_(5-11_1_-5) vacancies. The number of dangling bonds largely determines the relative stability of the layer with the same missing atoms. For example, the V_4_(5-5-5-9) structure is more stable than V_4_(5-12_2_-5), due to existence of two dangling bonds (the subscript in parenthesis) in the latter case. Our simulations also demonstrated that instantly removing a number of atoms from silicene causes the bending or rippling of the monolayer so as to considerably reduce its surface area. Indeed, the rippled structure decreases the energy of the systems and may stabilize vacancies in the silicene layer.

**Figure 2:**
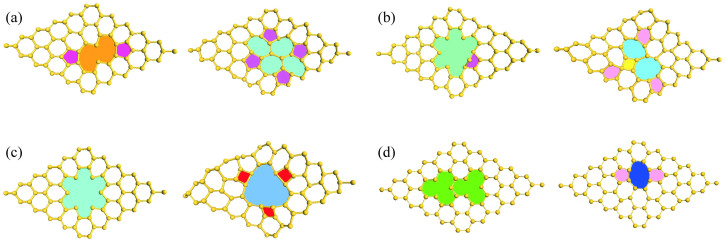
Snapshots from AIMD simulations at 500 K for the metastable (a) V_4_(5-12_2_-5), (b) V_5_(5-16_5_), and (c) V_6_(18_6_) vacancies at 0 ps (left) and 5 ps (right). The “re-reconstrucuted” configures are denoted as r-V_4_, r-V_5_, and r-V_6_, respectively. Plot (d) shows the two adjacent single vacancies coalesce into one V_2_(5-8-5) vacancy at 2 ps.

To assess whether the proposed vacancy defects in silicene is locally stable, we continued to perform *ab initio* molecular dynamics (AIMD) simulations on the reconstructed structures shown in Fig. 1. During a 5 ps AIMD run at 500 K, we observed that the metastable V_4_(5-12_2_-5), V_5_(5-16_5_), and V_6_(18_6_) structures destabilized and reconstructed again (“re-reconstructed”) to reduce all dangling bonds in the system (see Fig. 2). Importantly, these “re-reconstructed” structures remain less stable than the V_4_(5-5-9-9), V_5_(5-11_1_-5), and V_6_(5-5-10-5-5) shown in Fig. 1. For example, the computed formation energy of V_4_(5-5-5-9) is 0.09 and 0.70 eV lower than that of r-V_4_ and V_4_(5-12_2_-5), respectively, confirming the validity of our DFT-predicted vacancy structures. We now discuss the coalescence of small vacancies in silicene. The tendency of coalesce small defects into larger ones can actually be roughly estimated by the analysis of the *E_f_*′ values shown in Fig. 1. For example, the formation energy of two V_1_(5-5) is significantly larger than that of one V_2_(5-8-5), suggesting that divacancy is more preferable compared to two single vacancies in the silicene. Our AIMD runs at 500 K further confirmed the above hypothesis: two adjacent single vacancies indeed coalesce and transform to the V_2_(5-8-5) structure at 2 ps (see Fig. 2d).

**Figure 3:**
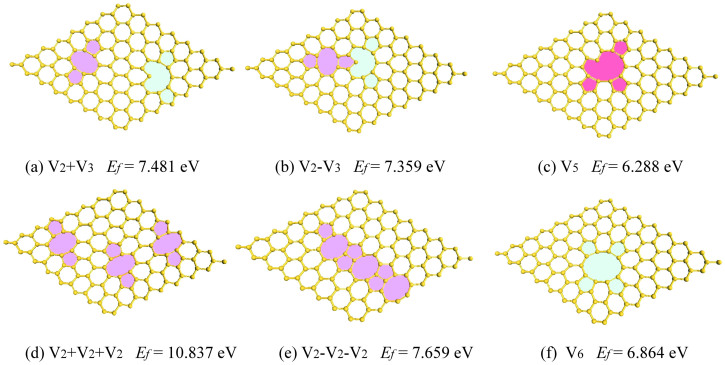
Relaxed structures of five- and six-atom vacancies in a (8 × 8) silicene supercell. The computed formation energies clearly indicate that the separated (a and d) and adjacent (b and e) defects are less stable than a single vacancy cluster (c and f).

The above conclusion, *i.e.*, small defects tend to coalesce into larger ones, can also be achieved for five-atom and six-atom vacancies. Figure 3 shows the six possible initial structures in the relatively larger (8 × 8) supercell. For 5-atom vacancies, we computed three configures: (1) V_2_ + V_3_, when a two-atom vacancy (V_2_) and a three-atom vacancy (V_3_) are separated [Fig. 3a]; (2) V_2_-V_3_, when the above two vacancies are adjacent [Fig. 3b]; and (3) V_5_, when a five-atom cluster is missing [Fig. 3c]. After optimization we found that V_5_ has the lowest energy whilst V_2_ + V_3_ is the most unstable structure, suggesting that the two separate defects are inclined to merge into a larger vacancy cluster. Similar findings can also be achieved in the case of 6-atom vacancies. As shown in Fig. 3, the three separated V_2_ defects have the highest energy, and when the three V_2_ defects are arranged in a line and adjacent, the energy decreased and then the three V_2_ defects coalesce into one six-atom cluster vacancies. The coalescence process in silicene is probably driven by the weak but long-ranged interactions between the vacancies, due to electronic effects^29^. Since long-range correlations are expected to be more important in anisotropic and low-dimensional structures^30^,^31^,^32^, we recalculated the *E_f_* values for the V_1_(5-5), V_2_(5-8-5), and V_6_(5-5-10) structures using the dispersion-inclusive DFT-D2 method^33^. For small vacancy clusters like V_1_(5-5), the *E_f_* values are almost identical no matter whether the van der Waals (vdW) interactions are taken into account in our DFT calculations. Notably, vdW forces contribute more to the bonding in larger vacancy clusters. As exemplified in the V_6_(5-5-10) structure, the computed formation energy from DFT-D2 is 8.4% larger than that from the PBE functional. However, our systematic tests have confirmed that the inclusion of the vdW energy does not change the relative stability of different defect systems.

### Structures and stability of extended line defect in silicene

**Figure 4:**
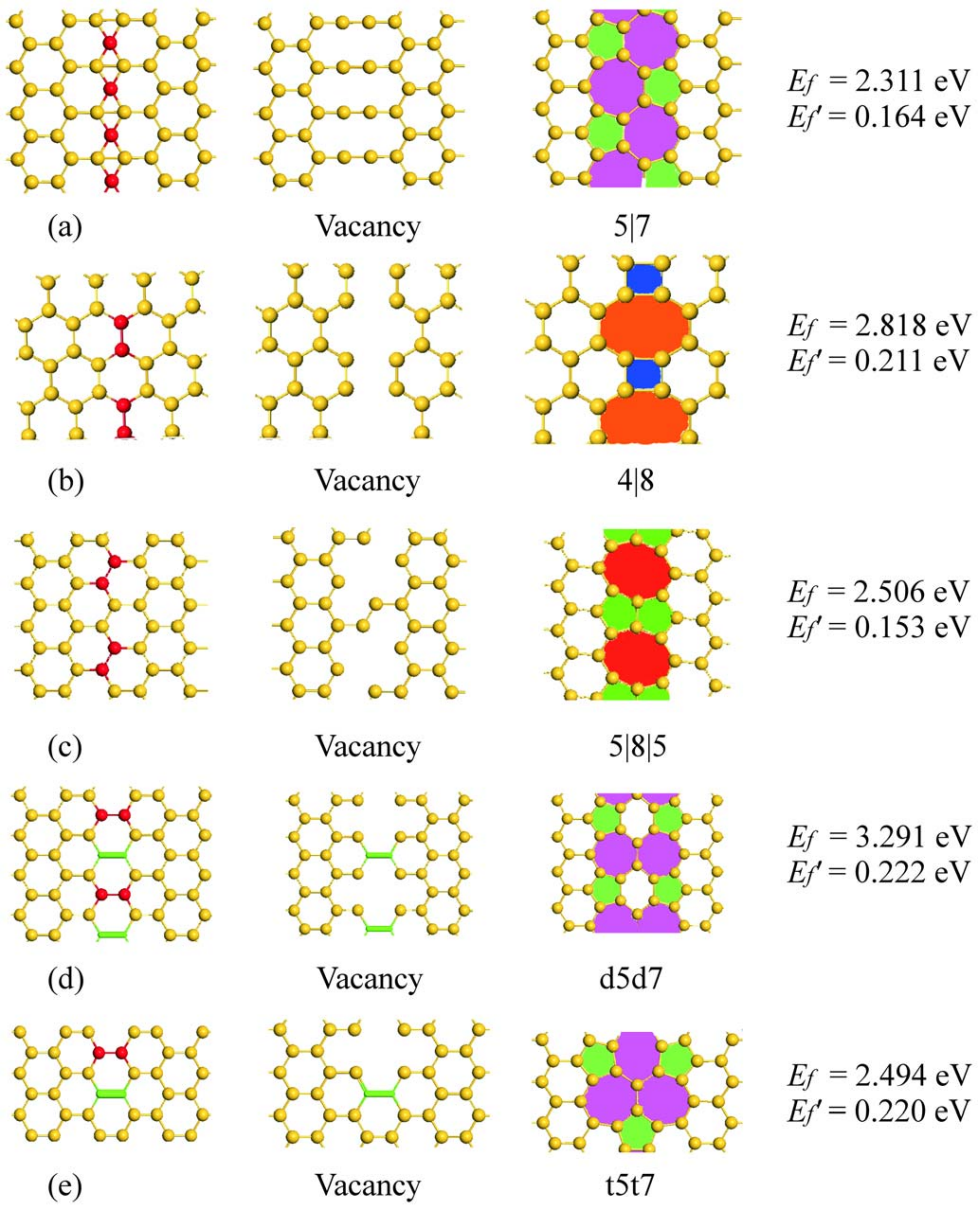
Formation of different extended lines of defects in silicene through the reconstruction of periodic vacancies. Lines of defects are formed after removing silicon atoms (in red) and rotating Si-Si bonds (in green).

ELDs in a two-dimensional (2D) lattice can be seen as a relative displacement of the two halves of the lattice, with possible removal of certain atoms located on or adjacent to the dislocation. In the case of silicene, the 5|7 and 4|8 ELDs are formed along the zigzag and armchair boundary, respectively (see Figs. 4a and 4b). After full relaxation, the neighboring half-lattices couple to each other and form periodic arrays of vacancies. The 5|8|5 ELD shown in Fig. 4c contains two pentagons spatially separated by an octagonal ring, which is obtainable by removing silicon dimmer stilted 30° from the zigzag chain.

In analog to graphene^34^,^35^,^36^, array of defects can be reconstructed from divacancies plus a Stone-Wales (SW) transformation. This leads to the formation of a double-pentagon double-heptagon structure (d5d7; Fig. 4d), and a triple-pentagon triple-heptagon structure (t5t7; Fig. 4e). Note that the above two ELDs, which can be obtained by irradiation, have been demonstrated to be stable topology for the reconstruction of an isolated divacancy in graphene^36^. Given that the buckled silicene requires a lower SW transformation barrier than graphene^24^, one would reasonably expect the existence of the d5d7 and t5t7 ELDs in the silicene monolayer.

The relative stability of ELDs in silicene can be estimated by the formation energy per unit, *E_f_*′, which is defined as
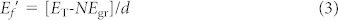
where *d* is length of the supercell along the ELD direction. Our calculations show that the 5|8|5 defect is the most stable structure, with a formation energy 0.06 eV/Å lower than that of the t5t7 defect. We have also compared the formation energy of the 5|8|5 ELD with the V_6_(5-5-10-5-5) structure shown in Fig. 1j – both having 6 vacancies in the (6 × 6) supercell – and found that the stability of the system can be dramatically enhanced when the vacancies are arranged in a line (3.59 *vs.* 6.63 eV for ELD and V_6_, respectively). In analog to graphene^35^, ELDs can significantly modify the electronic properties of silicene, which may transform semimetallic silicene into metallic.

### Adsorption and diffusion of silicon atoms on silicene

**Figure 5:**
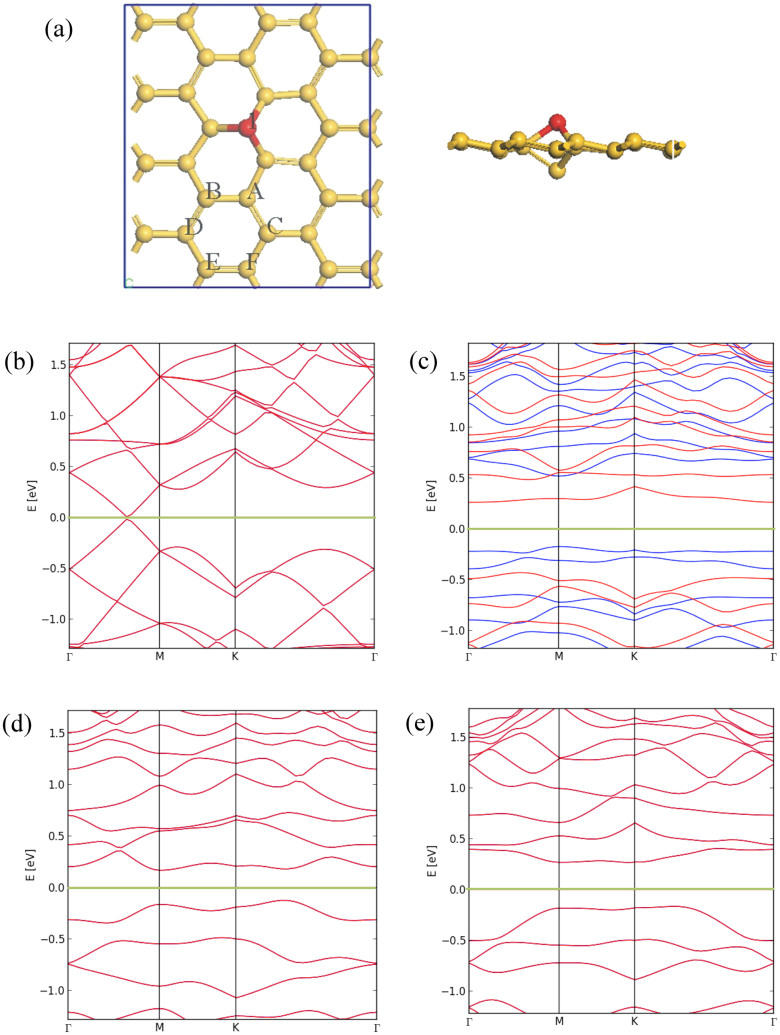
(a) Schematic plot of adsorption of Si adatoms at different sites on silicene sheet. Electronic band structures from the spin polarized HSE functional for (b) pristine silicene, (c) silicene with single adatom, (d) silicene with di-adatoms at sites 1 and B, (e) silicene with di-adatoms at sites 1 and C sites. Fermi level is set to zero. In plot (c), spin-up and spin-down bands are indicated by blue and red, respectively.

Adatoms are also an important defect of silicene. Consistent with previous studies^23^,^36^, our calculations found that Si adatoms prefer to adsorb at the top site of silicene, and forms the so-called “dumbbell configuration”^23^,^37^. Figure 5a shows the possible structures for co-adsorption of *two adatoms* on a silicene sheet, where the first Si is “fixed” at site 1, and the second one could stay at sites A to F. We define the adsorption energy between adatoms and silicene, *E*_ad_, as *E*_ad_ = (*E*_tot_-*E*_silicene_-*E*_si_)/*N*, where *E*_tot_, *E*_silicene_, and *E*_si_ denote the total energies of the entire system containing *N* adatoms, the pristine silicene, and an isolated silicon atom, respectively. The analysis of the *E*_ad_ and *E_f_* values shown in Table I indicates that the adsorption of adatoms on silicene is an exothermic process for all cases, and the systems are energetically more favorable than the pristine silicene when the second adatom locates at sites B to C. However, this is not the case when the two migrating adatoms are adjacent, *i.e.*, the second adatom stays at site A.

Table I also shows the computed energy barriers for Si adatoms diffusion on the silicene sheet, by using the LST/QST method^38^. Our calculations revealed that the diffusion of an adatom from A to B is significantly easier than to C (0.75 *vs.* 1.10 eV). Notably, the diffusion barriers for B→D and C→F are almost identical, but are nearly twice as large as that of the A→B pathway.

### Electronic and magnetic properties of adatom structures

Originating from the changes in localized states in sp^2^-bonded materials, adatoms would produce dispersion less metallic band or cause band-gap opening near the Fermi level. Suffering from the self-interaction errors, the standard DFT calculations are known to significantly underestimate the band gap of semiconductors. Moreover, recent work has demonstrated that semilocal generalized-gradient approximation (GGA) calculations would underestimate the relative stability of a spin-polarized configuration, and lead to an *incorrect* nonmagnetic ground state^39^. Thereby, we have employed the spin-polarized hybrid functional Heyd-Scuseria-Ernzerhof (HSE) for band structure calculations for the energetically favorable defect structures. As illustrated in Fig. 5b, the pristine silicene is a semimetal with the Dirac-like linear dispersion relationship, and its electronic structures can be significantly perturbed due to the adatom-induced symmetry-breaking effects. Indeed, upon the adsorption of a single Si atom at site 1, the Dirac cone vanishes and several bands near the Fermi level become almost flat, implying the strongly localized charge (see Figs. 5a and 5c). The HSE calculations for the single-adatom structure predict a spin-polarized ground state with a spin moment of 2 *μ*_B_, consistent with the literature^22^,^37^. Nevertheless, the band gap from the hybrid functional (0.40 eV) is significantly larger than that from GGA (0.08 eV)^37^. Further adsorption of the second Si atom leads to a considerably reduced band gap at site B (0.18 eV from HSE), a similar band gap at site C (0.36 eV), and a vanishing magnetic moment in both systems (see Figs. 5d and 5e). We also notice that all remaining di-adatom structures are metallic, with a spin moment of 2, 4, 0, and 4 *μ*_B_ at sites A, D, E, and F, respectively. We also checked the co-adsorption of three adatoms at sites 1, B, and F, which found to be the ground state with a spin moment of 2 *μ*_B_. Considering that adatom-induced structural deformation is significant in silicene, and the strain can be used to effectively tune their electronic band structures^40^, it is thus understandable that the electronic and magnetic properties of silicene monolayer can be modulated via controlling the number and the adsorption site of Si adatoms.

Comparing the thoroughly investigated C adatoms on graphene^41^,^42^ with Si adatoms on silicene studied in the present work, we see their differences and analogies in many aspects. For example, C adatoms prefer to adsorb to the bridge sites of graphene, whilst Si adatoms stay at the top site of silicene, along with a much larger binding energy in the latter case. On the other hand, the smallest energy barrier for C diffuses on graphene (0.74 eV) is close to that for Si diffuses from site A to B on silicene. Moreover, carbon adatoms also cause important change in electronic and magnetic properties, which can vary the spin moment of graphene from 0.12 to 0.27 μ_B_ at different coverages and adsorption sites^42^.

## Discussion

In summary, we theoretically studied the structure, energetics, and electronic properties of various defects in silicene, in the form of vacancy clusters, extended line defects, and di-adatoms. We have demonstrated that the vacancy defects in silicene tend to coalesce into a larger vacancy cluster and significantly stabilize the entire system. The formation energy of vacancy clusters can be dramatically decreased when defects are arranged in a line and formed ELDs, and the formation of the d5d7 and t5t7 ELDs in the silicene sheet is significantly easier than in graphene, due to the buckled silicene requires a lower SW transformation barrier than graphene. We also found that the presence of Si adatoms considerably enhances the stability of the pristine silicene, and more importantly, by changing the arrangement of two silicon atoms one can control the magnetic moment and open up the band gap in the silicene system.

## Methods

DFT calculations were carried out using the atomic-centered basis set SIESTA code^43^,^44^,^45^,^46^,^47^,^48^. The double-ξ pluspolarization orbitals (DZP) were adopted as atomic orbital basis sets, and the norm-conserving pseudo-potentials were constructed using the Trouiller and Martins scheme^48^. The charge density was projected on a real space grid with an equivalent cutoff 400 Ry to calculate the self-consistent Hamiltonian matrix elements. Geometry optimizations were performed with a residual force threshold of 0.01 eV/Å. The local spin-density approximation (LSDA)^43^ was used for vacancy defect calculations, which has been proven to give reasonable reconstructed structures and defect formation energies for 2D materials^35^,^36^. We used a (6 × 6) supercell, with a vacuum width of 30 Å, to assess the influence of various local defects in silicene. The configurations of silicene with defects were fully relaxed in terms of cell volume and the atomic coordinates with a conjugate gradient algorithm^49^. However, all angles between lattice vectors were constrained during relaxations. We used a Monkhorst-Pack^50^ k-point mesh of (8 × 8 × 1) for geometry optimization and (30 × 30 × 1) for electronic properties analysis. To avoid the interactions between imaging vacancy clusters, we used a larger (8 × 8) supercell in the study of coalescence for systems containing 5- and 6-atom vacancies.

## Author Contributions

S.L., W.L. and Y.Z. designed the research; S.L., Y.F.W., Y.T., Y.H.W. and T.J. performed calculations and prepared the figures; S.L. and W.L. wrote the paper.

## Figures and Tables

**Table 1:** The formation energy *E_f_* (in eV) and adsorption energy *E*_ad_ (eV/atom) for adatoms on silicene at different sites. The computed diffusion barriers (eV) for several pathways are also shown in the table

	Site	*E_f_*	*E*_ad_	Pathway	Barrier
One-adatom	1	−0.060	−3.862		
Two-adatoms	A	0.066	−3.768		
	B	−0.761	−4.182	A→B	0.75
	C	−0.599	−4.101	A→C	1.10
	D	−0.149	−3.876	B→D	1.35
	E	−0.261	−3.932		
	F	−0.024	−3.813	C→F	1.37

## References

[b1] Jose D. & Datta A. Structures and chemical properties of silicene: Unlike graphene. Acc. Chem. Res. 47, 593–602 (2014). 24215179 10.1021/ar400180e

[b2] Liu C. C., Feng W. & Yao Y. Quantum spin Hall effect in silicene and two-dimensional germanium. Phys. Rev. Lett. 107, 076802 (2011). 21902414 10.1103/PhysRevLett.107.076802

[b3] Xu M. S., Liang T., Shi M. M. & Chen H. Z. Graphene-like two-dimensional materials. Chem. Rev. 113, 3766–3798 (2013). 23286380 10.1021/cr300263a

[b4] Tsai W. F. *et al.* Gated silicene as a tunable source of nearly 100% spin-polarized electrons. Nat. Commun. 4, 1–6 (2013). 10.1038/ncomms2525 23422668

[b5] Ezawa M. Valley-polarized metals and quantum anomalous hall effect in silicene. Phys. Rev. Lett. 109, 055502 (2012). 23006186 10.1103/PhysRevLett.109.055502

[b6] Takeda K. & Shiraishi K. Theoretical possibility of stage corrugation in Si and Ge analogs of graphite. Phys. Rev. B 50, 14916–14922 (1994). 10.1103/physrevb.50.14916 9975837

[b7] Cahangirov S., Topsakal M., Akturk E., Sahin H. & Ciraci S. Two- and one-dimensional honeycomb structures of silicon and germanium. Phys. Rev. Lett. 102, 236804 (2009). 19658958 10.1103/PhysRevLett.102.236804

[b8] Ni Z. Y. *et al.* Tunable bandgap in silicene and germanene. Nano Lett. 12, 113–118 (2012). 22050667 10.1021/nl203065e

[b9] Gao N., Zheng W. T. & Jiang Q. Density functional theory calculations for two-dimensional silicene with halogen functionalization. Phys. Chem. Chem. Phys. 14, 257–261 (2012). 22083171 10.1039/c1cp22719j

[b10] Gao N., Li J. C. & Jiang Q. Bandgap opening in silicene: Effect of substrates. Chem. Phys. Lett. 529, 222–226 (2014).

[b11] Li S., Wu Y. F., Liu W. & Zhao Y. Control of band structure of van der Waals heterostructures: Silicene on ultrathin silicon nanosheets. Chem. Phys. Lett. 609, 161–166 (2014).

[b12] Li H. B. & Zhang R. Q. Vacancy-defect-induced diminution of thermal conductivity in silicene. EPL 99, 36001 (2012).

[b13] Vogt P. *et al.* Silicene: Compelling experimental evidence for graphene like two-dimensional silicon. Phys. Rev. Lett. 108, 155501(2012). 22587265 10.1103/PhysRevLett.108.155501

[b14] Feng B. J. *et al.* Evidence of silicene in honeycomb structures of silicon on Ag(111). Nano Lett. 12, 3507–3511(2012). 22658061 10.1021/nl301047g

[b15] Fleurence A. *et al.* Experimental evidence for epitaxial silicene on diboride thin films. Phys. Rev. Lett. 108, 245501(2012). 23004288 10.1103/PhysRevLett.108.245501

[b16] Meng L. *et al.* Buckled silicene formation on Ir(111). Nano Lett. 13, 685-690 (2013). 23330602 10.1021/nl304347w

[b17] Tchalala M. R. *et al.* Formation of one-dimensional self-assembled silicon nanoribbons on Au(110)-(2 × 1). Appl. Phys. Lett. 102, 083107 (2013).

[b18] Sadeghi H., Bailey S. & Lambert C. J. Silicene-based DNA nucleobase sensing. Appl. Phys. Lett. 104, 103104 (2014).

[b19] Brumfiel G. Sticky problem snares wonder material. Nature 495, 152–153 (2013). 23486035 10.1038/495152a

[b20] Banhart F., Kotakoski J. & Krasheninnikov A. V. Structural defects in graphene. ACS Nano 5, 26–41 (2011). 21090760 10.1021/nn102598m

[b21] Meyer J. C. *et al.* Direct imaging of lattice atoms and topological defects in graphene membranes. Nano. Lett. 8, 3582–3586 (2008). 18563938 10.1021/nl801386m

[b22] Guo J. F., Zhang J. F., Liu H. S., Zhang Q. F. & Zhao J. J. Structures, mobilities, electronic and magnetic properties of point defects in silicene. Nanoscale 5, 9785–9792 (2013). 23963524 10.1039/c3nr02826g

[b23] Özçelik V. O., Gurel H. H. & Ciraci S. Self-healing of vacancy defects in single-layer graphene and silicene. Phys. Rev. B 88, 045440 (2013).

[b24] Şahin H., Sivek J., Li S., Partoens B. & Peeters F. M. Stone-Wales defects in silicene: Formation, stability, and reactivity of defect sites. Phys. Rev. B 88, 045434 (2013).

[b25] Hu W., Xia N., Wu X. J., Li Z. Y. & Yang J. L. Helium separation via porous silicene based ultimate membrane. Nanoscale 5, 9062–9066 (2013). 23917262 10.1039/c3nr02326e

[b26] Ambrosetti A. & Silvestrelli P. L. Gas separation in nanoporous graphene from first principle calculations., *J. Phys*. Chem. C 118, 19172–19179 (2014).

[b27] Berdiyorov G. R. & Peeters F. M. Influence of vacancy defects on the thermal stability of silicene: A reactive molecular dynamics study. RSC Adv. 4, 1133–1137 (2014).

[b28] Jamgotchain H. *et al.* Silicene on Ag(111): Domains and local defects of the observed superstructures. J. Phys.: Conf. Ser. 491, 012001 (2014).

[b29] Kotakoski J., Krasheninnikov A. V. & Nordlund K. Energetics, structure, and long-range interaction of vacancy-type defects in carbon nanotubes: Atomistic simulations. Phys. Rev. B 74, 245420 (2006).

[b30] Ambrosetti A., Reilly A. M., Di Stasio Jr R. A. & Tkatchenko A. Long-rang correction energy calculated from coupled atomic response functions. J. Chem. Phys. 140, 18A508 (2014). 10.1063/1.4865104 24832316

[b31] Silvestrelli P. L. & Ambrosetti A. Including screening in van der Waals corrected density functional theory calculations: The case of atoms and small molecules physisorbed on graphene. J. Chem. Phys. 140, 124107 (2014). 24697424 10.1063/1.4869330

[b32] Gobre V. V. & Tkatchenko A. Scaling laws for van der Waals interactions in nanostructured materials. Nat. Commun. 4, 2341 (2013). 23955481 10.1038/ncomms3341 PMC3753541

[b33] Grimme S. Semi empirical GGA-type density functional constructed with a long-range dispersion correction. J. Comput. Chem. 27, 1787–1799 (2006). 16955487 10.1002/jcc.20495

[b34] Warner J. H., Lee G. D., Robertson A. W., Yoon E. & Kirkland A. I. Bond length and charge density variations within extended arm chair defects in graphene. ACS Nano 7, 9860–9866 (2013). 24148018 10.1021/nn403517m

[b35] Li Y., Zhang R. Q., Lin Z. J. & Van Hove M. A. Energetics and dynamics of a new type of extended line defects in graphene. Nanoscale 4, 2580–2583 (2012). 22434098 10.1039/c2nr30185g

[b36] Botello-Méndez A. R., Declerck X., Terrones M., Terrones H. & Charlier J. C. One-dimensional extended lines of divacancy defects in graphene. Nanoscale 3, 2868–2872 (2011). 21321755 10.1039/c0nr00820f

[b37] Özçelik V. O. & Ciraci S. Local reconstructions of siliceneinduced by adatoms. J. Phys. Chem. C 117, 26305–26315 (2013).

[b38] Halgren T. A. & Lipscomb W. N. The synchronous-transit method for determining reaction pathways and locating molecular transition states. Chem. Phys. Lett. 49, 225–232 (1977).

[b39] Kim H. J. & Cho J. H. Fluorine-induced local magnetic moment in graphene: A hybrid DFT study. Phys. Rev. B 87, 174435 (2013).

[b40] Mohan B., Kumar A. & Ahluwwalia P. K. Electronic and optical properties of silciene under uni-axial and bi-axial mechanical strain: A first principle study. Phys. E 61, 40–47 (2014).

[b41] Ataca C., Aktürk E., Şahin H. & Ciraci S. Adsorption of carbon adatoms to graphene and its nanoribbons, *J. App*. Phys. 109, 133704 (2011).

[b42] Zhou Y. G. *et al.* Electronic and magnetic properties of C-adsorbed graphene: A first-principles study. Phys. Chem. Chem. Phys. 13, 16574–16578 (2011). 21850307 10.1039/c1cp20482c

[b43] Hohenberg P. & Kohn W. Inhomogeneous electron gas. Phys. Rev. 136, B864–B871 (1964).

[b44] Kohn W. & Sham L. J. Self-consistent equations including exchange and correlation effects. Phys. Rev. 140, A1133–A1138 (1965).

[b45] Ceperley D. M. & Alder B. J. Ground state of the electron gas by a stochastic method. Phys. Rev. Lett. 45, 566–569 (1980).

[b46] Ordejon P., Artacho E. & Soler J. M. Self-consistent order-N density-functional calculations for very large systems. Phys. Rev. B 53, R10441–R10444 (1995). 10.1103/physrevb.53.r10441 9982701

[b47] Soler J. M. *et al.* The SIESTA method for ab initio order-N materials simulation. J. Phys: Consens. Matter 14, 2745–2779 (2002).

[b48] Troullier N. & Martins J. L. Efficient pseudopotentials for plane-wave calculations. Phys. Rev. B 43, 1993–2006 (1991). 10.1103/physrevb.43.1993 9997467

[b49] Press W. H., Flannery B. P., Teukolsky S. A. & Vetterling W. T. New Numerical Recipes (Cambridge University Press, New York,1986).

[b50] Monkhost H. J. & Pack J. D. Special points for Brillouin-zone integrations. Phys. Rev. B 13, 5188–5192 (1976).

